# Beneficial Bacteria in the Intestines of Housefly Larvae Promote Larval Development and Humoral Phenoloxidase Activity, While Harmful Bacteria do the Opposite

**DOI:** 10.3389/fimmu.2022.938972

**Published:** 2022-07-06

**Authors:** Qian Zhang, Shumin Wang, Xinyu Zhang, Kexin Zhang, Ying Li, Yansong Yin, Ruiling Zhang, Zhong Zhang

**Affiliations:** ^1^ School of Basic Medical Science, Shandong First Medical University (Shandong Academy of Medical Sciences), Taian, China; ^2^ The First Affiliated Hospital of Shandong First Medical University, Jinan, China

**Keywords:** housefly, gut bacteria, 16S rRNA, humoral immunity, phenoloxidase

## Abstract

The gut microenvironment of houseflies provides unique conditions for microbial colonization. Some gut microorganisms provide benefits for the development of the host by regulating the interaction between the host and intestinal pathogens. Gut microbial alterations can stimulate the host’s immune mechanism to resist pathogen invasion and affect the development of insects. In this study, we isolated 10 bacterial strains from housefly larval intestines. The isolated bacteria were added to the larval diet to analyze the effects of microecological regulation of gut bacteria on larval development. Dynamic changes in gut flora composition after oral administration of specific bacteria were analyzed although 16S rRNA gene high-throughput sequencing technology. To explore the interaction between gut bacteria and the host, the immune response of larvae against the invasion of foreign microorganisms was observed through a phenoloxidase activity experiment. Our results showed that the oral administration of various isolated bacteria had different effects on larval development. Oral administration of beneficial bacteria, including *Enterobacter hormaechei*, *Klebsiella pneumoniae*, *Acinetobacter bereziniae*, *Enterobacter cloacae*, *Lysinibacillus fusiformis* and *Bacillus safensis*, promoted larval development by increasing gut community diversity and the humoral immunity of larvae, while harmful bacteria, including *Pseudomonas aeruginosa*, *Providencia stuartii* and *Providencia vermicola*, influenced larval development by inhibiting the growth of beneficial bacteria and reducing the humoral immunity of larvae. The beneficial bacteria isolated in our research could be applied as good probiotic additives for the intensive feeding of larvae, while isolation of the harmful bacteria provides a basis for the development of pest inhibitors. Furthermore, our research revealed the immune response of housefly phenoloxidase to exogenous microorganism stimulation, providing richer and more comprehensive knowledge of the larval innate immune response.

## Introduction

As an important interface between the host and the external environment, there are a large number of microorganisms in the intestine, mainly bacteria, which are known as gut bacteria (1). In recent years, studies have found that gut bacteria play an important role in insect nutrient metabolism, development, behavior and defense against pathogen invasion. For example, *Blattabacterium*, as a Flavobacterial endosymbiont in cockroaches and termites (*Mastotermes darwiniensis*), can use nitrogen-containing organic wastes in essential amino acid and vitamin biosynthesis to provide nutrition for the host (2). Moreover, Enterobacteriaceae in the midgut of the Mediterranean fruit fly (*Ceratitis capitata*) express dinitrogen reductase and perform nitrogen fixation in the host (3). Additionally, *Serratia* strain Y1 inhibits *Plasmodium* development through activation of the mosquito immune system (4). Finally, *Chromobacterium* (Csp_P) can inhibit the growth of other bacterial species in the midgut of mosquitoes and colonize the midgut tissue to stimulate mosquito immune responses, which significantly reduces the survival of both the larval and adult stages (5).

When facing the invasion of extraneous pathogenic microorganisms, basic immune systems, including the PPO system (6), the Duox-ROS system and the IMD signaling pathway (7), play essential roles in helping insects maintain microbial homeostasis and resist pathogen invasion. Phenoloxidase (PO), which contains copper binding sites in animals, plants and insects, is one of the essential enzymes in the insect immune system to defend against microbial invaders. In recent years, Phenoloxidase has been widely studied. Phenoloxidase in insect humoral immunity exists in the epidermis, hemolymph and midgut in the form of prophenoloxidase (proPO) (8). When insects are invaded by foreign microorganisms, inactive PPO becomes activated PO under the action of related serine proteases and then forms quinones. PPO can regulate phagocytosis, coating and melanization to participate in the process of insect immune defense (9). PO participates in insect hemostasis and the immune defense response of foreign invaders and plays an extremely important role in insect growth, development, and immunological function (8, 10). The gene expression of *PPO1* and *PPO2* and the PO activity in the hemolymph of *Bombyx mori* reached peaks at 12 h after feeding on the gram-negative bacterium *Escherichia coli* and peaked at 24 h after feeding on the gram-positive bacterium *Staphylococcus aureus* (11). The *Blattella germanica* phenoleoxidase gene (BgPO) participates in the immune response to defend against *E. coli* invasion, and the PO activity of the hemolymph increases with increasing bacterial invasion time (12). Differences in the immunological and stress responses of *Spodoptera frugiperda* were observed after *E. coli* invasion (13). *Red palm weevil* gut flora can increase the survival rate and improve the immune capacity of larvae by upregulating important immune genes, indicating that the gut flora can stimulate the immune system of the RPW (14).

Houseflies, as health pests worldwide, are closely related to human beings and can spread a variety of diseases. A large number of microorganisms have been identified in the gut of houseflies, including *Klebsiella*, *Morganella*, *Providencia* and *Pseudomonas* (15). Our previous study showed that *Pseudomonas aeruginosa* strain Y12 in the gut of housefly larvae could protect the larvae from *Beauveria bassiana* invasion by the production of antifungal compounds (16). Different antibiotics inhibit the development of housefly larvae at the gene and microbiome levels (17). Houseflies, like other insects, can resist the invasion of various pathogens by the innate immune response. Previous studies have found that *E. coli* and *S. aureus* infection can cause strong melanization and activate the proPO system in *Musca domestica*, indicating that PO is involved in the immune system, helping host larvae defend against pathogenic microorganism invasion (18). However, there are few reports analyzing the effects of feeding isolated bacteria on the host gut community and the changes in PO activity in the hemolymph of larvae. This study explored the interaction between gut bacteria and houseflies through a feeding test and 16S rRNA gene high-throughput sequencing technology and analyzed the immune response of larvae against extraneous microorganism invasion through PO activity experiments. Beneficial or harmful bacteria were screened out, laying a theoretical foundation for the use of these organisms in the biological control of pest insects. More importantly, we explored the changes in larval PO activity to elucidate the innate immunity of housefly larvae.

## Methods

### Housefly Breeding

The housefly colony was reared in the Laboratory of Vector and Vector-borne Diseases of Shandong First Medical University since 2005. The housefly adults were fed with brown sugar and water, and the larvae were fed with wet wheat bran and milk powder [Wheat bran (g): DI water (mL): milk powder (g) = 1: 1: 0.25]. They were raised in an artificial climate incubator with a temperature of 25 ± 1°C and 70 ± 5% relative humidity with a photoperiod of 12/12 h (Light/Dark).

### Isolation of Gut Bacteria From Housefly Larvae

Bacteria were isolated according to the experimental method of previous studies (19). The normal reared housefly larvae were soaked in 75% alcohol for 10 minutes, cleaned with sterile double-distilled water for 3 times to eliminate surface impurities. Grind the sample thoroughly with an automatic grinder, coated on nutrient agar medium with 100ul each, placed in constant temperature incubator at 37°C for 24h until the bacteria colonies were formed. According to the difference of morphology and other characteristics of bacteria, separated and purified until a single colony was obtained. All the experimental operations were strictly aseptic.

### Effects of Feeding Isolated Bacteria on Larval Development

The isolated gut bacteria were inoculated into the Luria-Bertani liquid medium and placed in constant temperature culture oscillator at 37°C and 110 rpm/min for 24 h. The concentration of isolated bacteria in the gut reached 1x10^8^ cfu/mL, which were named Eh, Kp, Ya, Ab, Ps, Ec, Ll, Lf, Pv and Bs respectively, and were used in the feeding experiment.

The isolated bacteria in the larval were used as the experimental group, the Luria-Bertani liquid medium was the negative control group (named Lb), and the sterile water was the blank control group (named Wa). They were mixed with sterilized wheat bran in the ratio of 2:1 and stirred evenly. An equal amount of wheat bran was placed in a 10 mL centrifuge tube with small holes on the top to ensure air permeability, and 10 normal-breeding, good-growing and uniform-sized 1-day-old housefly larvae were fed with each tube, and a piece of gauze was placed between the tube and the lid to prevent the larvae from escaping. All housefly larvae were placed in an artificial climate incubator with a temperature of 25 ± 1°C, relative humidity of 70 ± 5%, and a photoperiod of 12/12 h (L/D). At predetermined time points (Day1, 2, 3, 4), the equal number of larvae were taken from each tube, and the body length, body weight and pupal weight were recorded, with three independent experiments of each group containing three replicates performed. After removing the surface debris, the larva samples were put into a 1.5 mL centrifuge tube, strictly disinfected and stored at - 80°C.

### Effects of Feeding Isolated Bacteria on the Gut Community Structure of the Larvae

The larvae samples were sent for 16S rRNA gene high-throughput sequencing, including Extraction of the intestine DNA, PCR amplification, Illumina MiSeq sequencing and bioinformatics analysis according to our previous studies (20). The larvae taken out of different treatment groups and control groups each time were used as a sampling unit, and each sampling unit had 3 replicates.

### Effects of Feeding Isolated Bacteria on Phenoloxidase Activity in Housefly Larvae

1-day-old housefly larvae were fed with different diets. At predetermined day (day1, 2, 3, 4), six uniform-sized housefly larvae of each group were collected. Add 0.5 mol/L Phosphate Buffer (pH = 7.0) to the larva sample, homogenize it in an ice bath, centrifuge at 4°C, 12000 r/min for 20 min, and the supernatant is the enzyme solution to be used to study the activity of phenoloxidase. According to the previous study (11). 3 ml of enzyme activity system was prepared, including 0.2 ml of enzyme solution, 1.3 ml of 0.2 mol/L Phosphate Buffer (pH = 6.8), 1.5 ml of 0.2 mol/L Catechol. The reaction was shaken in a 25°C water bath for 15 minutes, and the OD_600_ value was measured at the wavelength of 420 nm.

### Statistical Analysis

The experimental data was analyzed by Microsoft Excel 2010 and IBM SPSS 20 software. The effects of the body weight, body length and pupa weight of housefly larvae were compared by using one-way ANOVA followed by Sidak correction; The activity of phenoloxidase in haemolymph of the larvae was analyzed by Student’ s *t*-test. Asterisks indicate significant difference at ^*^ *P*<0.05, ^**^ *P*<0.01, ^***^ *P*<0.001.

## Results

### Isolation of Gut Bacteria From Housefly Larvae

The sequences amplified by 16S rRNA gene sequencing technology were aligned and analyzed on the NCBI website. Sequences with over 97% homology were used as references. A total of 10 bacteria were isolated from the guts of housefly larvae, including *Enterobacter hormaechei, Klebsiella pneumoniae, Pseudomonas aeruginosa, Acinetobacter bereziniae, Providencia stuartii, Enterobacter cloacae, Lactococcus lactis, Lysinibacillus fusiformis, Providencia vermicola* and *Bacillus safensis* (**Table 1**).

**Table 1:** Bacteria isolated from the guts of housefly larvae.

Number	Strain	Gen us	Homology
Yl	*Enterobacter hormaechei*	*Enterobacter*	99.30%
Y2	*Klebsiella pneumoniae*	*Klebsiella*	99.77%
Y3	*Pseudomonas aeruginosa*	*Pseudomonas*	100%
Y4	*Acinetobacter bereziniae*	*Acinetobacter*	97.50%
Y7	*Providencia stuartii*	*Providencia*	99.77%
Y8	*Enterobacter cloacae*	*Enterobacter*	99.53%
Y9	*Lactococcus lactis*	*Lactococcus*	99.88%
YI1O	*Lysinibacillus fusiformis*	*Lysinibacillus*	97.91%
Y11	*Providencia vermicola*	*Providencia*	99.42%
Y 1 2	*Bacillus safensis*	*Bacillus*	99.80%

### Effects of Feeding Isolated Bacteria on Larval Development

**Figure 1:**
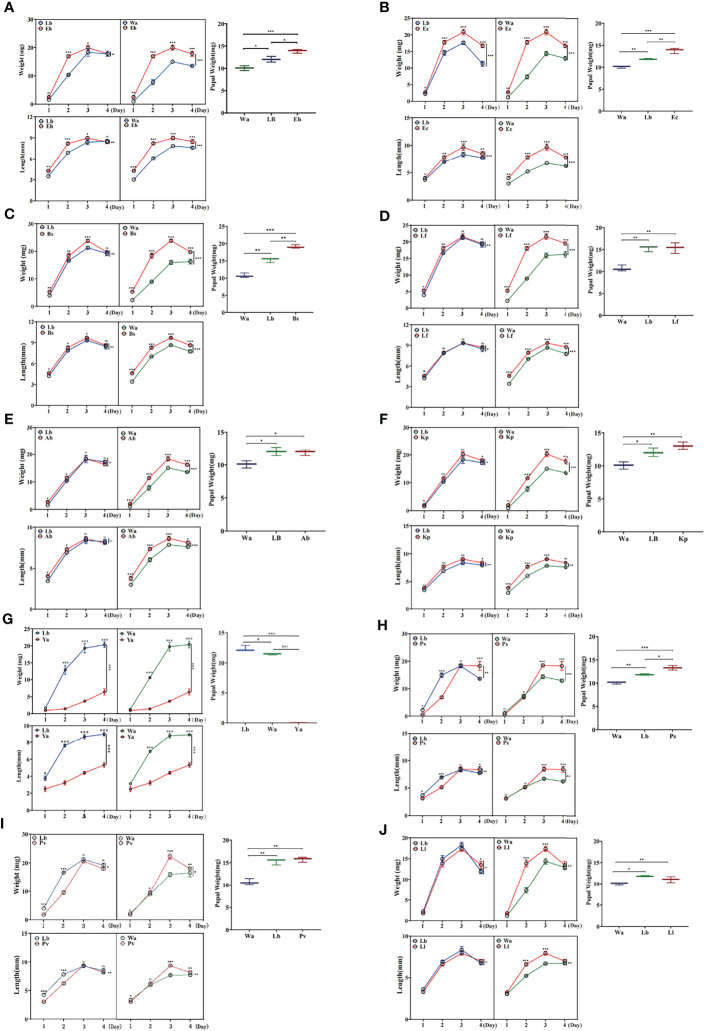
Effects of feeding isolated bacteria on larval development. Eh, Enterobacter hormaechei; Ec, Enterobacter cloacae; Bs, Bacillus safensis; Lf, Lysinibacillus fusiformis; Ab, Acinetobacter bereziniae; Kp, Klebsiella pneumoniae; Ya, Pseudomonas aeruginosa; Ps, Providencia stuartii; Pv, Providencia vermicola; Ll, Lactococcus lactis; Wa, sterile water, blank control group; Lb, Luria-Bertani liquid medium, negative control group; Day 1, Day 2, Day 3, Day 4: the developmental period of housefly larvae. Pupal weight was measured on the first day they pupated. *p < 0.05, **p < 0.01, ***p < 0.001.

The effects of isolated bacteria on the development of larvae were identified by feeding experiments. The results showed that the body weight and body length of larvae in the Eh, Ec, Bs and Kp groups increased significantly compared with those of the negative control group (Lb) and the blank control group (Wa) (**Figures 1A–C, F**), and the body weight and body length of larvae increased significantly in Lf1d, Lf2d, Ab1d and Ab2d compared with the negative control group (Lb) and the blank control group (Wa) (**Figures 1D, E**). Increases in body length and body weight of larvae in the Ya groups were significantly inhibited compared with those of the negative control group (Lb) and the blank control group (Wa) (**Figure 1G**), and the increases in body weight and body length of the larvae were significantly inhibited in Ps1d, Ps2d, Pv1d and Pv2d compared with the negative control group (Lb) and the blank control group (Wa) (**Figures 1H, I**). Compared with the negative control group (Lb), there was no significant difference in body weight or body length of housefly larvae in the Ll group (**Figure 1J**). Moreover, compared with the control group (Lb), the pupal weight increased in the Eh, Ec, Bs and Ps groups, and there was no difference in pupal weight in the Kp, Ab, Lf, Ll and Pv groups. There was no pupation of housefly larvae in the Ya group (**Figure 1**). The effects of the isolated bacteria on the pupation and emergence rate of the housefly larvae in other groups were further analyzed. Our results revealed that pupation and emergence rate of the housefly larvae in group Eh, Kp, Ec and Bs increased compared to the control group (**Table 2**). In conclusion, *E. hormaechei, K. pneumoniae, A. bereziniae, E. cloacae, L. fusiformis* and *B. safensis* can promote the growth and development of housefly larvae and were classified as “beneficial bacteria” in the guts of the larvae; *P. aeruginosa, P. stuartii* and *P. vermicola* can inhibit the growth and development of larvae and were classified as “harmful bacteria” in the guts of the larvae; *L. lactis* had no significant effect on the development of housefly larvae and was classified as a “neutral bacterium”.

**Table 2:** The impact of bacterial isolates on the pupation rate and emergence rate of the housefly.

	Lb	Wa	Eh	Kp	Ec	Bs	Ab	Lf	Pa	Ps	Pv	Ll
Pupation rate	76.00%	66.67%	90.67%	81.33%	86.67%	84.00%	78.67%	77.33%	0.00%	77.33%	74.67%	72.00%
Emergence rate	86.03%	83.98%	91.16%	88.57%	89.18%	88.87%	88.20%	87.96%	0.00%	84.81%	87.66%	85.26%

### Effects of Feeding Isolated Bacteria on the Gut Community Structure of the Larvae

**Figure 2:**
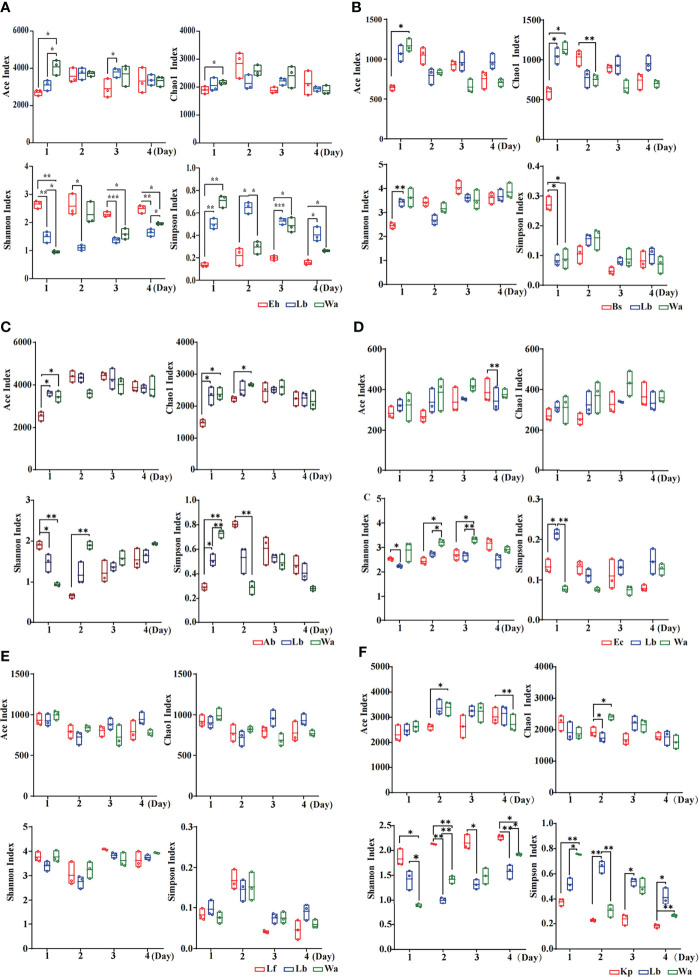
Effects of “beneficial bacteria” in the guts of housefly larvae on the intestinal diversity index of larvae. Eh, *Enterobacter hormaechei*; Ab, *Acinetobacter bereziniae*; Bs, *Bacillus safensis*; Ec, *Enterobacter cloacae*; Lf, *Lysinibacillus fusiformis*; Kp, *Klebsiella pneumoniae*. Wa, sterile water, blank control group; Lb, Luria-Bertani liquid medium, negative control group; Day 1, Day 2, Day 3, Day 4: the developmental period of housefly larvae. *p < 0.05, **p < 0.01, ***p < 0.001.

**Figure 3:**
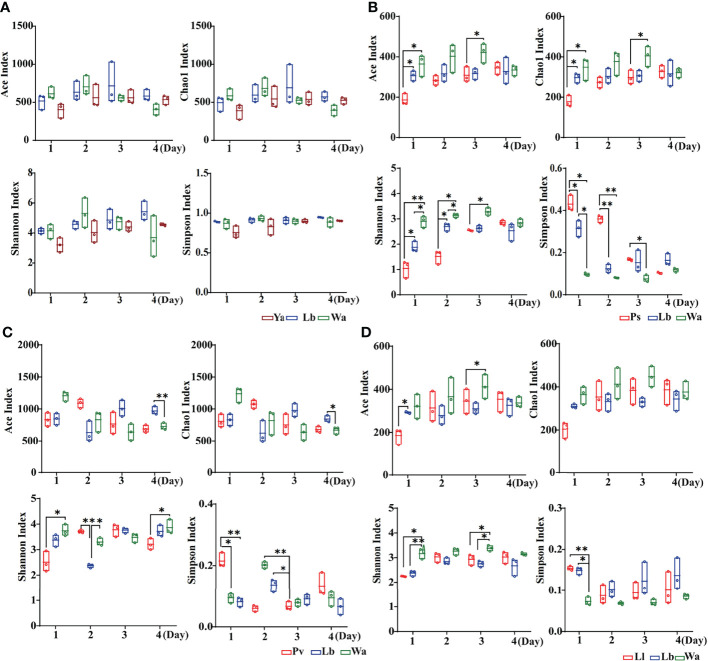
Effects of “harmful bacteria” **(A–C)** and “neutral bacteria” **(D)** in the guts of housefly larvae on the intestinal diversity index of larvae. Ya, *Pseudomonas aeruginosa*; Ps, *Providencia stuartii*; Pv, *Providencia vermicola*; Ll, *Lactococcus lactis*. Wa, sterile water, blank control group; Lb: Luria-Bertani liquid medium, negative control group; Day 1, Day 2, Day 3, Day 4: the developmental period of housefly larvae. *p < 0.05, **p < 0.01, ***p < 0.001.

The Ace index and Chao1 index were used to represent the richness of larval gut flora, and the Shannon index and Simpson index were used to represent the diversity of larval gut flora. After feeding housefly larvae with “beneficial bacteria”, the gut flora richness of housefly larvae decreased in Eh1d, Bs1d and Ab1d, there was no significant difference in gut flora richness in Ec and Kp; the gut flora diversity of larvae increased in Eh, Kp, Ab and Ec (**Figures 2A, C, D, F**), while the gut flora diversity of larvae decreased in Bs (**Figure 2B**), and there was no significant difference in the gut flora index in Lf (**Figure 2E**). After feeding housefly larvae with “harmful bacteria”, there was no significant difference in the gut flora index in Ya (**Figure 3A**), the gut flora index of housefly larvae decreased in Ps (**Figure 3B**), and the gut flora diversity of housefly larvae decreased in Pv1d (**Figure 3C**). After feeding housefly larvae with “neutral bacteria”, there was no significant difference in gut flora richness in Ll, and the gut flora diversity of housefly larvae decreased in Ll1d (**Figure 3D**).

**Figure 4:**
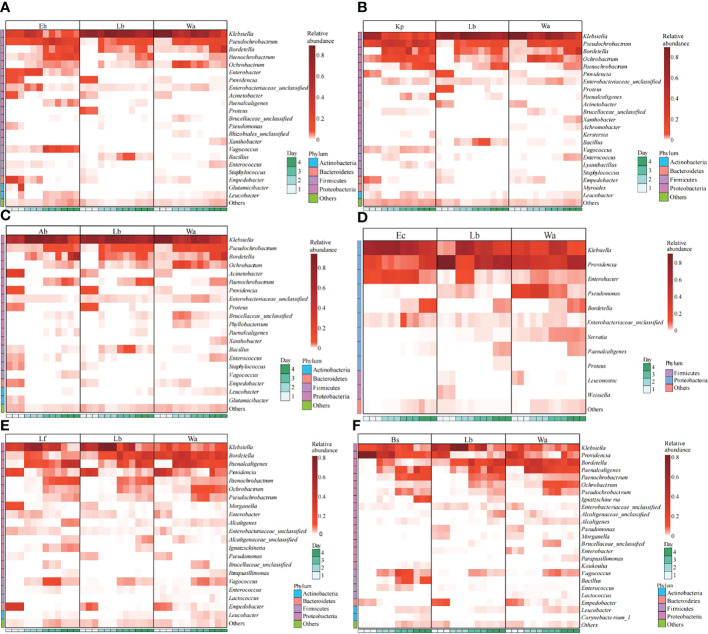
Effects of “beneficial bacteria” in the guts of housefly larvae on the composition of gut flora of larvae. Eh, *Enterobacter hormaechei*; Ec, *Enterobacter cloacae*; Bs, *Bacillus safensis*; Lf: *Lysinibacillus fusiformis*; Ab, *Acinetobacter bereziniae*; Kp, *Klebsiella pneumoniae*. Wa, sterile water, blank control group; Lb, Luria-Bertani liquid medium, negative control group; Day 1, Day 2, Day 3, Day 4: the developmental period of housefly larvae.

The regulatory mechanism by which the isolated bacteria impact the gut flora of the larvae was analyzed by 16S rRNA gene sequencing technology. The results showed that different bacteria isolated from housefly larvae had different effects on the larval gut flora. After feeding housefly larvae with “beneficial bacteria”, the abundance of *Enterobacter, Acinetobacter, Empedobacter* and *Pseudomonas* in the gut bacterial composition of the larvae increased in Eh1d and Eh2d, the abundances of *Bordetalla, Paenochrobactrum, Paenalcaligenes, Vagococcus* and *Leucobacter* increased in Eh3d and Eh4d, and the abundance of *Klebsiella*, *Proteus* and *Bacillus* decreased (**Figure 4A**). The abundance of *Paenalcaligenes*, *Ochrobactrum, Bordetalla, Paenochrobactrum* and *Vagococcus* increased in Kp2d, Kp3d and Kp4d, and the abundance of *Klebsiella, Acinetobacter* and *Bacillus* decreased (**Figure 4B**). The abundance of *Acinetobacter, Enterococcus, Empedobacter* and *Staphylococcus* in Ab1d increased; the abundance of *Bacillus* and *Pseudochrobactrum* decreased in Ab2d (**Figure 4C**). The abundance of *Enterobacter*, *Klebsiella* and Bordetella in the gut bacterial composition of the larvae in the Ec group increased; the abundance of *Providencia*, *Pseudomonas* and *Serratia* decreased (**Figure 4D**). The abundance of *Providencia* and *Pseudomonas* decreased in Lf1d, while the abundance of *Empedobacter* and *Morganella* increased in Lf1d. The abundance of *Bordetella* and *Ochrobactrum* decreased in Lf3d and Lf4d, while the abundance of *Paenochrobactrum, Vagococcus* and *Ignatzschineria* increased in Lf3d and Lf4d (**Figure 4E**). The abundance of *Bacillus*, *Paenochrobactrum, Vagococcus, Providencia, Ignatzschineria* and *Enterococcus* increased in Bs, and the abundance of *Empedobacter, Morganella* and *Enterobacter* decreased in Bs1d (**Figure 4F**). Therefore, the abundance of *Empedobacter* increased after feeding *E. hormaechei, L. fusiformis* and *A. bereziniae*. The abundance of *Paenochrobactrum* and *Vagococcus* increased after feeding *E. hormaechei, B. safensis, K. pneumoniae* and *L. fusiformis*. The abundance of *Ignatzschineria* increased after feeding *B. safensis* and *L. fusiformis*. However, the abundance of *Bacillus* decreased after feeding *E. hormaechei, K. pneumoniae* and *A. bereziniae*, and the abundance of *Providencia* decreased after feeding *E. hormaechei, E. cloacae, L. fusiformis* and *A. bereziniae.*

**Figure 5:**
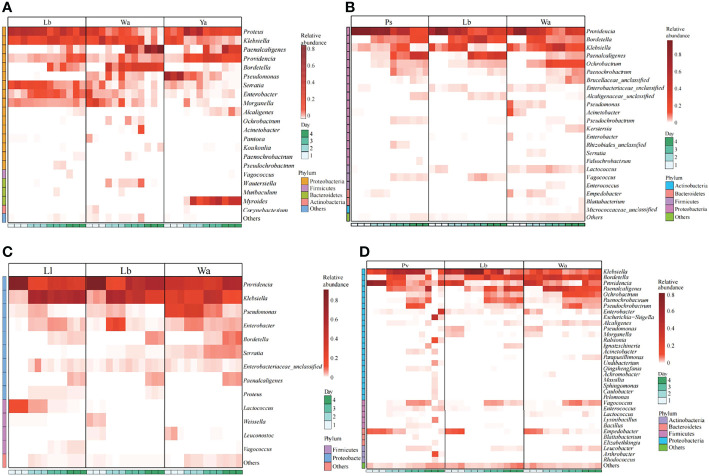
Effects of “harmful bacteria” **(A–C)** and “neutral bacteria” **(D)** in the guts of housefly larvae on the composition of gut flora of larvae. Ya, *Pseudomonas aeruginosa*; Ps, *Providencia stuartii*; Pv, *Providencia vermicola*; Ll, *Lactococcus lactis*. Wa, sterile water, blank control group; Lb: Luria-Bertani liquid medium, negative control group; Day 1, Day 2, Day 3, Day 4: the developmental period of housefly larvae.

After feeding housefly larvae with “harmful bacteria”, the abundance of *Pseudomonas* in Ya1d and Ya2d increased significantly, the abundance of *Providencia, Proteus, Myroides* and *Alcaligene*s increased significantly in Ya2d, Ya3d and Ya4d, while the abundance of *Klebsiella, Bordetella, Morganella, Serratia* and *Enterobacter* decreased significantly in Ya2d, Ya3d and Ya4d (**Figure 5A**). The abundance of *Providencia* and *Paenochrobactrum* increased, and the relative abundance of *Klebsiella* decreased in Ps1d and Ps2d. The relative abundance of *Bordetalla* decreased in Ps2d and Ps3d, while the abundance of *Pseudomonas* decreased in Ps1d and Ps2d (**Figure 5B**). The abundance of *Providencia* increased in Pv1d and Pv2d, the abundance of *Klebsiella* decreased in Pv1d and Pv2d, and the abundance of *Bordetella* and *Ochrobactrum* decreased in Pv3d and Pv4d (**Figure 5C**). Therefore, the abundance of *Providencia* increased and the abundance of *Bordetella* and *Klebsiella* decreased after feeding *P. aeruginosa, P. stuartii* and *P. vermicola*.

After feeding housefly larvae with “neutral bacteria”, compared with the control group, the abundance of *Lactococcus* increased in Ll1d and Ll2d, and the abundance of *Vagococcus* and *Klebsiella* increased in Ll3d and Ll4d (**Figure 5D**).

### Effects of Feeding Isolated Bacteria on Phenoloxidase Activity in Housefly Larvae

**Figure 6:**
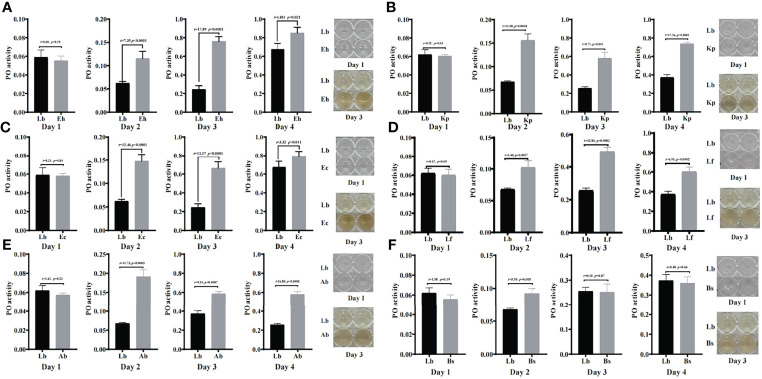
Effects of “beneficial bacteria” in the guts of housefly larvae on phenoloxidase activity in the hemolymph. Eh, *Enterobacter hormaechei*; Ec:, *Enterobacter cloacae*; Bs, *Bacillus safensis*; Lf, *Lysinibacillus fusiformis*; Ab, *Acinetobacter bereziniae*; Kp, *Klebsiella pneumoniae*. Day 1, Day 2, Day 3, Day 4: the developmental period of housefly larvae.

**Figure 7:**
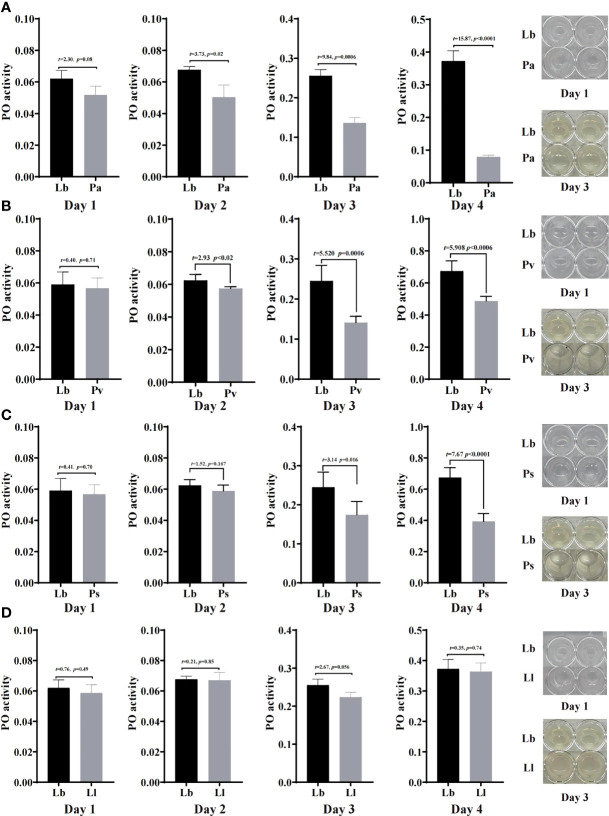
Effects of “harmful bacteria” **(A–C)** and “neutral bacteria” **(D)** in the guts of housefly larvae on phenoloxidase activity in the hemolymph. Ya, *Pseudomonas aeruginosa*; Ps, *Providencia stuartii*; Pv:, *Providencia vermicola*; Ll, *Lactococcus lactis*. Day 1, Day 2, Day 3, Day 4: the developmental period of housefly larvae.

To study the immune response by which houseflies resist the invasion of pathogenic bacteria, the effects of the isolated bacteria on phenoloxidase activity in the larval hemolymph were analysed. The results showed that there was no significant difference in phenoloxidase activity or melanization ability in the hemolymph after the larvae were fed gut bacteria for 1d compared with that in the control group (*P*>0.05) (**Figures 6**, **7**).

After feeding housefly larvae with “beneficial bacteria” for 3 days, compared with the control group, the phenoloxidase activity and melanization in the hemolymph of housefly larvae in the Eh, Kp, Ec, Lf and Ab groups increased significantly (*P*<0.05) (**Figures 6A–E**). The phenoloxidase activity in the hemolymph of the larvae increased significantly in Bs2d (*P*<0.05), while the melanization ability in the hemolymph of the larvae increased in Bs2d, Bs3d and Bs4d (**Figure 6F**).

After feeding housefly larvae with “harmful bacteria” for 3 days, compared with the control group, the activity of phenoloxidase in the hemolymph of the larvae increased significantly in Ya and Pv, and there was no melanization in the hemolymph of the larvae (**Figures 7A, B**). The activity of phenoloxidase in the hemolymph of the larvae was significantly inhibited in Ps3d and Ps4d, and there was no melanization in the hemolymph of the larvae (**Figure 7C**).

After feeding housefly larvae with “neutral bacteria” for 3 days, compared with the control group, there was no significant difference in phenoloxidase activity in the hemolymph of the Ll larvae. Moreover, the results also showed that the activity of phenoloxidase in the hemolymph of housefly larvae increased gradually with the development of the larvae (**Figure 7D**).

## Discussion

In this study, 10 bacteria were isolated from the guts of housefly larvae, including *Enterobacter*, *Providence*, *Pseudomonas*, *Lactococcus*, *Klebsiella*, *Bacillus* and *Acinetobacter*. The composition of gut bacteria of the larvae was mostly consistent with the results reported in our previous research, and the differences may have been caused by the differences in feeding conditions and larval sources (21). Through the feeding experiment, it was found that the growth index of housefly larvae fed with Luria-Bertani liquid medium was significantly higher than that of flies fed with sterile water because the Luria-Bertani liquid medium contained artificially prepared nutrients for the growth of microorganisms, insects and animals, which had a beneficial impact on the development of housefly larvae, while sterile water was only used to maintain the lives of the larvae. By comparisons with the negative control and the blank control, we can better understand the regulatory effect of gut bacteria on larval development.

According to the influence of feeding isolated bacteria on the development and the composition of gut flora of housefly larvae, the isolated bacteria in the gut of the larvae can be roughly divided into three categories, mainly including “beneficial bacteria” that can promote the development of housefly larvae, including *E. hormaechei*, *E. cloacae*, *K. pneumoniae*, *B. safensis*, *A. bereziniae* and *L. fusiformis*. Furthermore, 16S rRNA gene high-throughput sequencing technology revealed that the abundance of gut microorganisms in the larvae changed differently after feeding the “beneficial bacteria”; for example, the abundance of *Empedobacter*, *Vagococcus*, *Ignatzschineria*, *Acinetobacter* and *Enterococcus* increased, and the abundance of *Bacillus, Klebsiella* and *Providencia* decreased. Studies have shown that metabolites of *Bacillus cereus* can lure massive amounts of *Bactrocera dorsalis* adults, providing insight for the development of bacterial biocontrol agents and the production of insecticides (22). *Enterococcus* has stronger environmental persistence than *E. coli* and is considered to be a robust organism capable of withstanding various environmental pressures (23). We speculate that changes in these genera enhance the stability of gut flora and the adaptability of larvae to the surrounding environment, leading to favorable development of larvae. “Beneficial bacteria” in the guts of housefly larvae can act as probiotic supplements for larvae, participate in metabolism and provide nutrients for the host to improve the development and utilization of insect resources. The addition of *Enterobacter* sp. changed the bacterial load of Enterobacteriaceae in the intestinal tract of *Bactrocera cucurbitae*, reduced the abundance of *Pseudomonas* and significantly improved the quality control parameters of the flies (24). Probiotic bacteria (*Klebsiella pneumonia*, *Enterobacter* spp. and *Klebsiella oxytoca*) increased the number of Enterobacteriaceae in the intestines of *Ceratitis capitata* and improved the quality control parameters and sexual function of male flies (25). “Harmful bacteria” that can inhibit the development of housefly larvae included *P. aeruginosa*, *P. stuartii* and *P. vermicola*. 16S rRNA gene sequencing technology revealed that the abundance of *Providencia* increased and the abundance of *Bordetella* decreased in the gut flora of the larvae after feeding “harmful bacteria”. We speculated that *P. aeruginosa*, *P. stuartii* and *P. vermicola* can inhibit the development of larvae by increasing the number of harmful bacteria and reducing beneficial bacteria. “Harmful bacteria” in the gut of housefly larvae can be developed into housefly growth inhibitors, which provides science-guided ideas for pest control strategies. In the Mediterranean fruit fly (*Ceratitis capitata*), a high concentration of *Pseudomonas aeruginosa* in the gut can reduce the longevity of the host (26). The death rate of the host and the level of immune response of the host are different after *Providencia sneebia* and *Providencia rettgeri* infection with *Drosophila melanogaster* (27). “Neutral bacteria” that have no effect on the development of housefly larvae included *L. lactis*, which can maintain the growth of larvae by increasing the number of *Lactococcus* in the guts of larvae and assisting the gut flora. *L. lactis* does not play a major role in the growth of housefly larvae but plays a certain role in maintaining the balance of gut flora. *Lactobacillus* can promote the absorption of feed, increase the nutritional value of feed and improve the growth performance of animals (28). *Lactobacillus* can enhance the activity of animal immune cells to resist the invasion of pathogens and improve immunity and resistance to diseases (29).

At present, phenoloxidase activity is used as the standard index to evaluate the immune ability of insects (30). Through the phenoloxidase activity experiment, we found that “beneficial bacteria” in the gut of housefly larvae promoted phenoloxidase activity and melanization ability in the hemolymph of the larvae after feeding, indicating that “beneficial bacteria” in the guts of housefly larvae can not only promote larval development but also improve their humoral immunity. “Harmful bacteria” in the gut of the larvae inhibited the phenoloxidase activity and the melanization ability in the hemolymph of the larvae after feeding, indicating that “harmful bacteria” in the guts of housefly larvae can not only hinder their development but also inhibit their humoral immunity. “Neutral bacteria” in the gut of housefly larvae had no significant effect on the phenoloxidase activity in the hemolymph of the larvae after feeding. The above results show that different bacteria may activate different pattern recognition receptors after invading the larvae, which may have different effects on the activation of the PPO system and finally lead to different effects on the phenoloxidase activity and melanization ability in insect hemolymph. Because the 1-day-old housefly larvae were small and the phenoloxidase activity was weak, there was no significant difference in the phenoloxidase activity between the experimental group and the control group. The 3-day-old housefly larvae have a thinner body wall and the highest phenoloxidase activity, which improves the immunity of the larvae. With the development of larvae, the body wall of larvae gradually hardens, resulting in a decrease in phenoloxidase activity in the larvae. These results are similar to the distribution of phenoloxidase activity in the hemolymph of *Bombyx mori* (31). *E. coli* and *S. aureus* infection induced stronger melanization ability in housefly larvae than in pupae and adults (18). However, we found that oral administration of harmful bacteria can reduce the phenoloxidase activity in housefly larvae. We assume that intake of harmful bacteria affected the gut flora and reduced the increase in beneficial bacteria, which negatively influenced the larval humoral immunity and the response ability of insects to the external environment. In summary, the bacteria isolated from the guts of housefly larvae and the developmental period of larvae had a certain impact on the phenoloxidase activity in the hemolymph of larvae. The study of the effects of gut bacteria on melanization and phenoloxidase activity of houseflies also lays a good foundation for further study on the mechanism of the PPO system of houseflies in the future. Moreover, the innate immune response is a way for insects to resist pathogens. Using gut microbiota to affect host immunity can be considered a very promising pest management strategy.

## Conclusion

**Figure 8:**
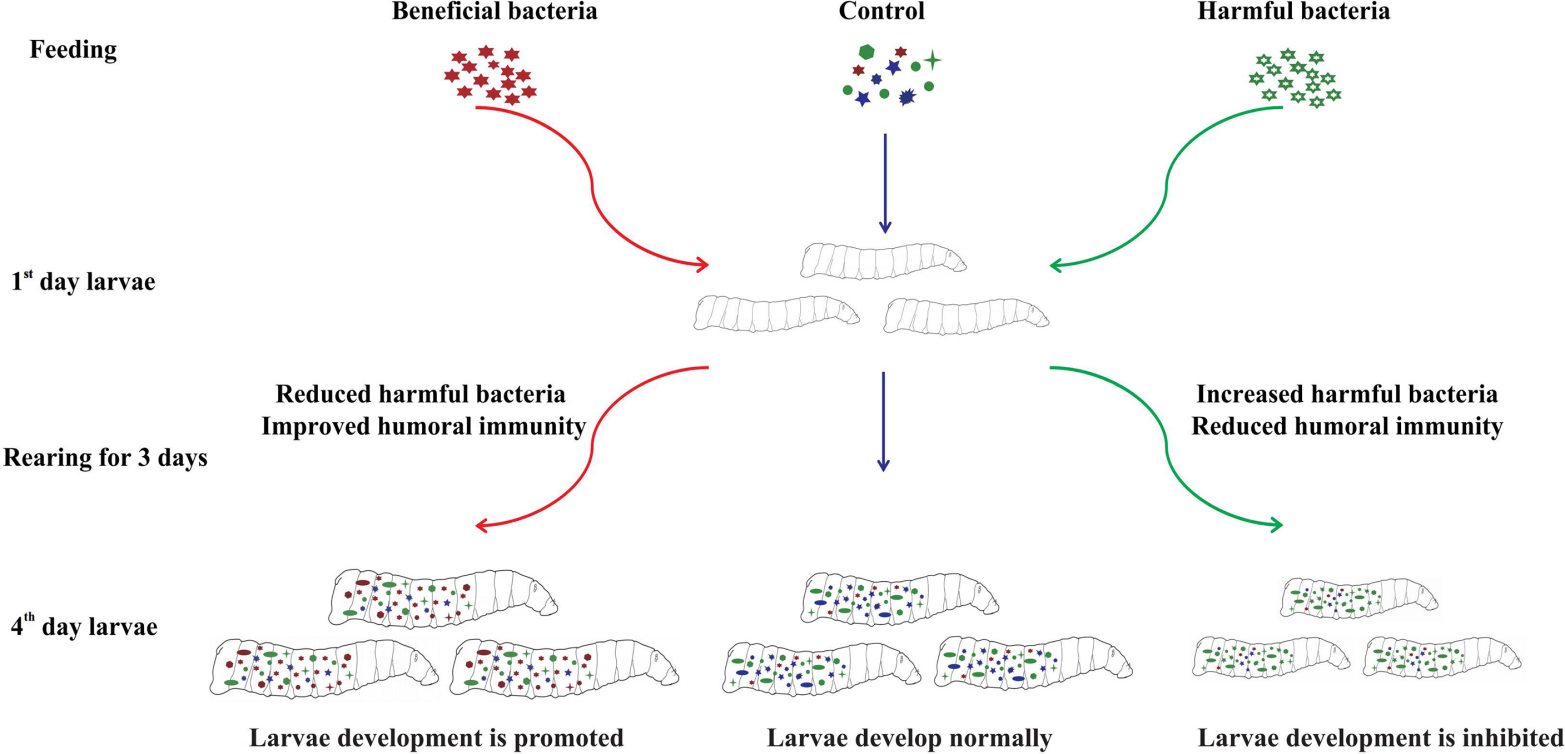
Schematic diagram of the effects of novel isolated bacteria on housefly development.

Oral administration of various isolated bacteria had different effects on the gut community structure and phenoloxidase activity of housefly larvae, which further affected larval development (**Figure 8**). Feeding “beneficial bacteria” to housefly larvae can inhibit the growth of some “harmful bacteria” in the intestine, increase the number of “beneficial bacteria” in the gut, improve the humoral immunity of larvae, and finally promote larval development. Feeding “harmful bacteria” to housefly larvae can inhibit the growth of some “beneficial bacteria” in intestines, resulting in an abnormal increase in the number of “harmful bacteria”, a disorder of the gut community structure and the reduction of the humoral immunity of larvae, which finally inhibits the growth of larvae. Therefore, the dynamic stabilization of gut microbiota is necessary for larval development. Alteration of the gut community structure influences larval innate immunity and larval growth.

## Data Availability Statement

The datasets presented in this study can be found in online repositories. The name of the repository and accession number can be found below: NCBI Sequence Read Archive; PRJNA725070.

## Author Contributions

RZ and ZZ conceived and directed the project together. QZ, SW, XZ, KZ, YL and YY performed the experiments. QZ and SW analyzed the results and wrote the manuscript. RZ and ZZ revised the manuscript. All authors contributed to the article and approved the submitted version.

## Funding

This work was supported by the National Natural Science Foundation of China (No.81572028 and 81871686).

## Conflict of Interest

The authors declare that the research was conducted in the absence of any commercial or financial relationships that could be construed as a potential conflict of interest.

## Publisher’s Note

All claims expressed in this article are solely those of the authors and do not necessarily represent those of their affiliated organizations, or those of the publisher, the editors and the reviewers. Any product that may be evaluated in this article, or claim that may be made by its manufacturer, is not guaranteed or endorsed by the publisher.
